# European youth care sites serve different populations of adolescents with cannabis use disorder. Baseline and referral data from the INCANT trial

**DOI:** 10.1186/1471-244X-11-110

**Published:** 2011-07-12

**Authors:** Olivier Phan, Craig E Henderson, Tatiana Angelidis, Patricia Weil, Manja van Toorn, Renske Rigter, Cecilia Soria, Henk Rigter

**Affiliations:** 1 Centre Emergence, Institut Mutualiste Montsouris, Paris, France; Inserm U669; Université Paris-Sud et Paris Descartes; UMR-S0669; Paris, France; 2 Department of Psychology, Sam Houston State University, Huntsville, Texas, USA; 3 Department of Psychiatry, CHU Brugmann, Université Libre de Bruxelles, Brussels, Belgium; 4 Delphi-Gesellschaft für Forschung, Berlin, Germany; 5 Parnassia Addiction Research Centre, The Hague, the Netherlands; 6 Fondation Phénix, Geneva, Switzerland; 7 Department of Public Health, Erasmus MC, Rotterdam, the Netherlands; 8 Department of Child and Adolescent Psychiatry, LUMC, Leiden, the Netherlands

## Abstract

**Background:**

MDFT (Multidimensional Family Therapy) is a family based outpatient treatment programme for adolescent problem behaviour. MDFT has been found effective in the USA in adolescent samples differing in severity and treatment delivery settings. On request of five governments (Belgium, France, Germany, the Netherlands, and Switzerland), MDFT has now been tested in the joint INCANT trial (International Cannabis Need of Treatment) for applicability in Western Europe. In each of the five countries, study participants were recruited from the local population of youth seeking or guided to treatment for, among other things, cannabis use disorder. There is little information in the literature if these populations are comparable between sites/countries or not. Therefore, we examined if the study samples enrolled in the five countries differed in baseline characteristics regarding demographics, clinical profile, and treatment delivery setting.

**Methods:**

INCANT was a multicentre phase III(b) randomized controlled trial with an open-label, parallel group design. It compared MDFT with treatment as usual (TAU) at and across sites in Berlin, Brussels, Geneva, The Hague and Paris.

Participants of INCANT were adolescents of either sex, from 13 through 18 years of age, with a cannabis use disorder (dependence or abuse), and at least one parent willing to take part in the treatment. In total, 450 cases/families were randomized (concealed) into INCANT.

**Results:**

We collected data about adolescent and family demographics (age, gender, family composition, school, work, friends, and leisure time). In addition, we gathered data about problem behaviour (substance use, alcohol and cannabis use disorders, delinquency, psychiatric co-morbidity).

There were no major differences on any of these measures between the treatment conditions (MDFT and TAU) for any of the sites. However, there were *cross-site *differences on many variables. Most of these could be explained by variations in treatment culture, as reflected by referral policy, i.e., participants' referral source. We distinguished 'self-determined' referral (common in Brussels and Paris) and referral with some authority-related 'external' coercion (common in Geneva and The Hague). The two referral types were more equally divided in Berlin. Many cross-site baseline differences disappeared when we took referral source into account, but not all.

**Conclusions:**

A multisite trial has the advantage of being efficient, but it also carries risks, the most important one being lack of equivalence between local study populations. Our site populations differed in many respects. This is not a problem for analyses and interpretations if the differences somehow can be accounted for. To a major extent, this appeared possible in INCANT. The most important factor underlying the cross-site variations in baseline characteristics was referral source. Correcting for referral source made most differences disappear. Therefore, we will use referral source as a covariate accounting for site differences in future INCANT outcome analyses.

**Trial registration number**

ISRCTN: ISRCTN51014277

## Background

In 1999, the (junior) Ministers of Health of Belgium, France, Germany, the Netherlands, and Switzerland agreed that their countries were disputing each other's cannabis policies without having enough data to support any stance. They wished to combine scientific efforts. The Five-Countries Action Plan for Cannabis Research from April 2003 stressed the need of a transnational trial to test an outpatient treatment of cannabis use disorder and associated problems (e.g., delinquency, psychiatric co-morbidity) among youth in the five Western European countries mentioned [1]. The treatment chosen for this trial on the basis of its record of empirical support was Multidimensional Family Therapy (MDFT), developed by Liddle and colleagues at the Center for Treatment Research on Adolescent Drug Abuse (CTRADA), University of Miami Miller School of Medicine [2]. The study was named INCANT (INternational CAnnabis Need for Treatment). It is a randomized controlled trial (RCT) comparing MDFT with treatment as usual (TAU) at and across sites in Berlin, Brussels, Geneva, The Hague and Paris.

MDFT is a family based outpatient treatment programme for adolescent problem behaviour [1 - 4]. Key to MDFT is the assumption that each major domain in the life of an adolescent may contribute to the incidence and persistence of behavioural problems (through risk factors) and may help in resolving such problems (through protective factors). The life domains include the youth itself, parent, family, friends and peers, school and work, and leisure time. In 5 to 7 months, the therapist carries out, in rapid succession, therapy sessions with the adolescent alone, with the parents alone, with the family (youth and parents), and sometimes with representatives from systems outside the family (friends, school, probation office, etc.) present. The therapist sets out to improve life domain conditions for the adolescent and the family in an outreaching fashion. MDFT views family functioning as instrumental in creating new, developmentally adaptive lifestyle alternatives for the adolescent. Skills training includes substance use relapse prevention, family communication, and parenting.

MDFT has been tested with success in different adolescent populations, doses and treatment delivery settings in the USA [3,4].

Hurdles had to be overcome before the INCANT trial could start. A RCT was controversial in Western European youth care at the time. It was feared that a standardized (manual-based) time-limited treatment like MDFT would not be accepted in France, with its dominant psycho-analytic treatment tradition or in Germany (Berlin), where treatment of substance abusing adolescents often lasted for more than 1 year. Further, Swiss clinicians believed that coercing adolescents into treatment, which was deemed feasible in INCANT, would fail to convince cases to accept or complete treatment. Nevertheless, we managed to mount the INCANT study.

The process of randomization ensures that study groups - i.e., the MDFT and TAU groups - are equivalent on baseline characteristics. CONSORT, which is the opinion leading Consolidated Standards of Reporting Trials group, finds it illogical, but not wrong, to test for statistically significant differences between trial groups at baseline, because by definition any difference found is due to chance rather than the result of a factor causing variation between groups (http://www.consort-statement.org). However, this does not apply to multisite trials such as INCANT. INCANT succeeded in randomizing study participants on a number of stratification variables, but one set of variables could not be included in the randomization process: the local treatment culture in a city/country, and the local referral and other treatment-related policies. Therefore, we performed statistical analyses to assess the INCANT sites from the five countries on comparability of study participants' baseline characteristics.

Issues like these increasingly turn up in the treatment research literature, with its growing emphasis on 'practice-relevant studies'. Relevance for practice means that studies need to include sites with potentially different ways of delivering services due to varying local or national culture [5,6]. One of the primary methodological challenges facing such multisite trials is how to deal with site differences, and variability in treatment effects across sites. In this paper, we follow well-grounded recommendations from the literature for exploring baseline differences across sites in clinically-relevant background characteristics, as well as in variables to be used for primary and secondary outcome analyses [7,8].

## Methods

### Study design

INCANT was a multicentre phase III(b) randomized controlled trial with an open-label, parallel group design. This study compared MDFT with TAU at and across sites in Berlin, Brussels, Geneva, The Hague and Paris. Part of TAU in Paris was specified in a treatment manual and was called TAU-e (e = explicit). In this paper, we combine TAU-e and TAU under the term 'TAU'; the distinction between the two treatment variants will feature in other publications. Assessments were carried out at baseline (immediately before randomization) and at 3, 6, 9 and 12 months after randomization. Before the trial started, INCANT was approved by all relevant ethical boards [1].

### Treatment centres

On the basis of a pilot study testing the feasibility of training European therapists in MDFT and the applicability of MDFT in European practice, the following treatment centres were selected for taking part in INCANT [1]. In Belgium, the Cannabis Clinic associated with the department of psychiatry of Brugmann University Hospital in Brussels was chosen, and in France the Centre Emergence in Paris, with suburban CEDAT (Conseils Aide et Action contre le Toximanie) sub-sites in Mantes la Jolie and St Germain en Laye. In Germany, Therapieladen in Berlin was selected. The Netherlands was represented by the twinning sites of Parnassia Brijder (Mistral, youth addiction care) and De Jutters (Palmhuis, youth forensic care) in The Hague. The Swiss site was Phénix in Geneva.

### Participants

Candidates for INCANT were adolescents of either sex, from 13 through 18 years of age, with a cannabis use disorder (dependence or abuse), and at least one parent willing to take part in the treatment. The word 'parent' denotes any legal representative of the adolescent, including step or foster parent, or guardian. We use the singular 'parent' here, also including the plural 'parents'.

Adolescents were ineligible if they had an IQ lower than 70, or were unable to understand the local language, unable to attend outpatient sessions, or if suffering from a mental or behavioural disorder that required inpatient treatment.

### Power calculations

The government representatives of the five European countries subsidizing the study requested INCANT to be one transnational trial rather than a collection of five local trials. The representatives wished to stimulate across-border research collaboration. To render across-site comparisons possible, each INCANT site adopted the same study procedures (informed consent, measurement instruments, assessments, etc.). A second reason why we opted for the 1 joint trial model followed from power calculations. According to our computations [1], each site needed to recruit 100 cases for an effect size difference between MDFT and TAU of d = 0,7 and power level of 0.82 (120 cases for power level 0.88). The Belgian and Swiss governments did not have sufficient funds to have 120 cases recruited in their countries. They settled for N = 60 each, explicitly signing in on across-site statistical analyses.

The recruitment target set for INCANT as a whole was 450 cases (= adolescents and their families) [1]. Brussels and Geneva opted for N = 60 each, Berlin and Paris for 120 each, and The Hague for 150.

### Recruitment and randomization

Procedures for baseline assessments, recruitment and concealed randomization have been described before [1]. Baseline assessments were conducted by research staff at each site, who had been trained in adhering to the three INCANT Instruction Manuals and whose performance was monitored by Erasmus MC and discussed in joint telephone meetings.

Randomization took place immediately after having obtained informed consent, with equal portions to be assigned to MDFT and TAU (1:1), except for Paris where the ratio between MDFT and TAU (including TAU-e) was roughly 1:2. In Berlin, Brussels, Geneva and Paris, we stratified the local study sample using three dichotomous variables (gender; age [13-14 years vs. 15-18 years]; and level of cannabis use in the past 90 days [74 or fewer days of cannabis consumption vs. 75 or more]). In The Hague, we added the stratification variable 'ethnic background' to the variables just mentioned. Across sites and sub-sites, there were 72 strata. For each stratum, the database computer generated 50 independent randomisations.

All sites except Paris had two randomisation arms (MDFT vs. TAU), and we used block randomisation with randomly permuted blocks of 2 or 4 cases. For Paris, with three randomisation arms, we used blocks of 3 or 6 cases.

**Figure 1:**
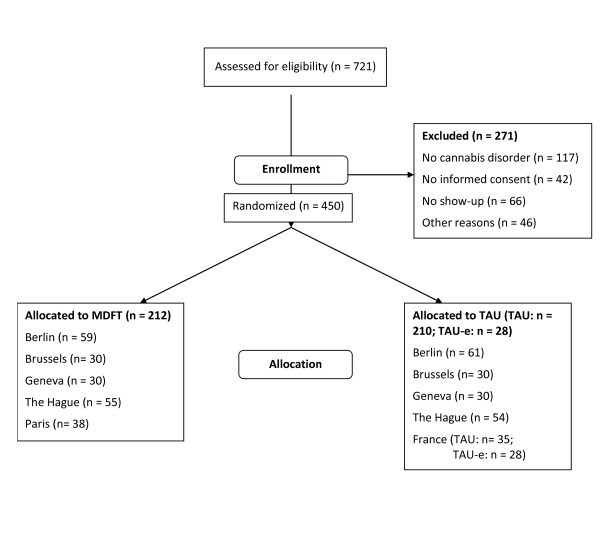
**INCANT recruitment flowchart**.

Across sites, we assessed 721 families for eligibility for the trial (Figure 1). Of these families, 271 (38%) were excluded, for reasons explained below. Not in the figure and not discussed here are 13 TAU cases in Paris who were not randomized into the trial but did take part in study surveys to learn more about TAU.

Baseline assessment was scheduled in two meetings, allowing the family time to consider giving informed consent in between the assessments. Cases were excluded if they failed to show up for the second meeting (66 cases; see Figure 1).

There were three other reasons for exclusion: (1) adolescents appearing to have no recent diagnosis of cannabis use disorder as examined at the second meeting (16% of all those assessed), (2) cases (adolescent and/or parent) refusing to sign informed consent (6% of cases assessed), and (3) 'other reason': these were mostly cases where the referral agency refused to accept treatment allocation to be randomized, or where the youth disappeared from sight before randomization occurred (e.g., because of detention or moving away).

Among all youth assessed, there were 604 adolescents with a cannabis use disorder. Of the latter group, 450 (75%) were enrolled in the study together with their parents. Pre-set recruitment targets were attained in Berlin, Brussels, Geneva and Paris. The Hague remained under its recruitment target of 150, because of staff health problems that prevented full operation for some time.

The excluded cases were similar to the included ones in age, gender and level of cannabis use (*p*s > 0.05).

### Central database

Each site had one or two researchers authorized to access their own site's internet based location - part of the Erasmus MC managed INCANT central database [1]. Only the Erasmus MC database manager had full access to all locations and was mandated to change inputted data if so instructed by the project leader (HR) on behalf of the international committee overseeing the design and execution of the trial.

### Measures

Measurement instruments were questionnaires and structured interviews. They were applied at baseline and at four follow-up assessment points [1].

#### Background and demographic information

The *Parent and Adolescent Interviews *[1] were used to collect demographic data on gender, age and ethnicity, and on family composition, history of family drug use and mental health problems, adolescent substance use history and court involvement, treatment history and service utilization, school functioning, peer relationships, and pastime activities.

#### Substance use

Youth were assessed for cannabis use and other substance use disorders in various ways. Most relevant here is the *Timeline Follow-Back *method (TLFB; validated for adolescents) [9]. The TLFB retrospectively recorded daily cannabis use for the 90-day period before baseline and other assessments, using a calendar and other memory prompts to stimulate recall.

Cannabis use and other substance use disorders were assessed with the *Adolescent Diagnostic Interview-Light *(ADI-Light; [1]). This brief structured, multi-axial interview is based on DSM-IV criteria for substance use disorders in adolescents.

#### Psychosocial functioning

We measured adolescents' symptoms of internalizing and externalizing disorders with the *Youth Self Report *(YSR) scales for Anxiety/Affective problems and for Aggression/Delinquency problems, respectively. The YSR has been proven to be reliable and valid across languages and countries, both at total instrument level [10,11] and at the level of the scales used in INCANT [12 - 14]. For the same items, we also administered the 'parent version' of the YSR, i.e., the CBCL (*Child Behavior Checklist *[15]).

#### Baseline measures also to be used as outcome measures

Most questionnaires and interviews were administered at more than one, or even all all assessment points, but in this paper we focus on the TLFB, YSR and CBCL.

#### Referral

We recorded by whom the case had been referred to the INCANT site for treatment. We distinguished six referral routes, viz. (1) self-referral (the adolescent took the initiative to contact the site him- or herself), (2) referral by relatives, friends or acquaintances, (3) by school, (4) by other treatment and care agencies, and (5) referral by Justice (youth probation officer or appointed family guardian, public prosecutor, court). When analyzing the data, we noted that referral source could be dichotomized into a binary variable distinguished by two classes of referral, i.e., Self-Determined (SD) and Externally Coerced (EC). Self-Determined is defined here as seeking referral on one's own accord or with some supportive (non-coercive) prompting by people from the adolescent's social environment. EC is any referral the adolescent feels he or she cannot resist out of fear of sanctions, such as being kicked out of something (school, services, and programmes), being placed out of home, or being detained or otherwise being sanctioned by Justice authorities.

The scientific committee overseeing INCANT, the IST, agreed on an algorithm to classify referral source as SD or EC. SD were all cases that were 'self-referred' or 'referred by relatives, friends, or acquaintances'. By definition, all Justice-related referrals were EC.

We decided to re-examine the cases referred by school or by another treatment or care agency. In our algorithm, referral by school was considered SD when referral carried no threat of the youth being sent away from school if refusing to accept treatment. Referral by another treatment or care agency was considered to be EC when some sanction was pending if treatment was refused. In The Hague, for instance, there were cases where the adolescent had been mandated by Justice to a mental health centre, which referred the adolescent on to the INCANT site, but with the original legal threat still lingering on (so, EC). Of the Berlin teenagers, one in five lived in a residential setting, including sheltered living (Betreutes Wohnen, i.e., the adolescent getting his own apartment and some pocket money, with supervision from a social worker). The pressure put on the adolescents in Betreutes Wohnen to seek help was considered EC.

For each 'school referred' and 'other treatment and care agency referred' adolescent, we asked the local researcher who had done the baseline assessment and the therapist who had given the treatment, to classify the case as SD or EC on the basis of the algorithm. There were no differences in opinion. All cases could be classified as either SD or EC. The project leader (HR) reviewed all these cases and found no reason to question the verdicts.

### Analyses

In countries with more than 1 sub-site (France and the Netherlands), we pooled the data at site level.

Analysis of Variance (ANOVA) was used to compare the treatments on continuous variables such as level of cannabis consumption, and *χ^2 ^*to compare them on categorical variables such as gender. *Post hoc *comparisons for the significant ANOVA models were conducted with the Tukey test. We also carried out a multivariate analysis of variance (MANOVA) for three intended outcome measures, using continuous data, pulled together.

Analyses were performed both across and within sites. Within sites, there were no significant statistical differences between treatment groups, and these data are not reported here (please contact the corresponding author for these results if desired). The results from statistical analyses reported below are from the cross-site analyses.

As missing data were rare at baseline (typically less than 1% per item), they were handled with list-wise deletion as proposed by Allison [16].

## Results

### Cross-site comparisons on the stratification variables

Were sites comparable on the stratification variables (age, gender, and level of cannabis consumption) at baseline? Table 1 presents an overview.

**Table 1:** Scores on stratification variables by treatment condition and site

**Variable**	***Brussels***		***Paris***		***Berlin***		***The Hague***		***Geneva***	
	***MDFT***	***TAU***	***MDFT***	***TAU***	***MDFT***	***TAU***	***MDFT***	***TAU***	***MDFT***	***TAU***
Aged 13 - 14 years	3%	3%	11%	5%	10%	12%	11%	9%	10%	10%
Male gender	93%	93%	92%	86%	81%	84%	80%	80%	90%	93%
Mean number of days of cannabis use	68 (21)	67 (23)	60 (25)	63 (27)	58 (28)	62 (24)	64 (23)	61 (24)	47 (25)	52 (29)

Figures between brackets = standard deviations

The average age of all INCANT adolescents was 16.3 years (standard deviation: 1.2), with no statistically significant difference between sites. For stratification, we distinguished a young age group (13 to 14 years of age) and an older one (15 through 18). Roughly one out of ten youth recruited were in the younger age category, irrespective of site and treatment condition (Table 1).

Of all adolescents, 86% were boys. There was a slight difference between sites, with Berlin and The Hague having lower proportions of boys than the other sites (*χ*^2 ^[4, 450] = 9.9, *p *= 0.04).

The TLFB was used to record days of cannabis consumption in the 90 days before the baseline assessment. Sites varied on the TLFB measure (ANOVA, *F *[4, 444] = 4.2, *p *= 0.002), with participants in Geneva reporting fewer days of cannabis use than participants in Brussels, The Hague, and Paris.

### Cross-site comparisons on other baseline characteristics: adolescents

Table 2 lists a number of baseline characteristics on which we compared treatment conditions. There were no significant differences within sites. We focus here on the comparisons *between *sites.

**Table 2:** Baseline characteristics of INCANT adolescents by site and treatment condition

**Variable**	***Brussels***		***Paris***		***Berlin***		***The Hague***		***Geneva***	
	***MDFT***	***TAU***	***MDFT***	***TAU***	***MDFT***	***TAU***	***MDFT***	***TAU***	***MDFT***	***TAU***
Living with family	97%	96%	100%	100%	79%	68%	98%	98%	82%	83%
Foreign descent	47%	27%	32%	34%	33%	25%	46%	48%	73%	60%
Cannabis dependence	97%	93%	79%	75%	86%	89%	73%	78%	90%	97%
Alcohol use disorder	67%	50%	34%	27%	66%	53%	18%	13%	57%	67%
Arrested in past 90 days	43%	40%	37%	44%	17%	23%	26%	30%	53%	47%
Mean internalizing symptoms	16.6 (8.0)	12.3 (6.9)	12.2 (10.5)	13.9 9.6)	16.3 (9.8)	17.3 (10.8)	14.1 (10.5)	13.7 (9.2)	13.0 (8.3)	14.4 (9.3)
Mean externalizing symptoms	23.1 (8.3)	19.4 (6.9)	19.4 (10.9)	17.3 (7.7)	23.8 (7.9)	22.5 (8.6)	19.7 (9.3)	17.6 (7.8)	21.7 (9.4)	22.7 (8.8)
In school	80%	80%	84%	89%	66%	67%	77%	74%	67%	70%
Employment (regular + temporary jobs)	63%	73%	14%	5%	21%	17%	62%	65%	26%	31%

Figures between brackets = standard deviations

#### Demographics: living with family

The vast majority of adolescents were still living with family, i.e., their parents or other relatives. Sites did not differ in this respect.

#### Demographics: foreign descent

An adolescent was considered to be from foreign descent if at least one of his or her parents had been born abroad. Sites differed in the proportion of adolescents with foreign background (*χ*^2 ^[4, 440] = 28.4, *p *< 0.001), with the highest proportion seen in Geneva, followed by The Hague (Table 2).

From which countries did the parent(s) of the youth with foreign background come from? Most dominant in Brussels were 'other European country' (55% of all those with foreign descent) and 'Africa' excluding North Africa (27%). For Paris, most prominent were 'North Africa' (52%) and 'other European country' (29%). In Berlin, 'other European country' (49%) prevailed among the nations of origin, with 'Turkey' (17%) at second place. The top two for The Hague were 'Surinam/Dutch Antilles' (60%) and 'North Africa' (13%; in particular Morocco). The sizable proportion of adolescents from foreign descent in Geneva was mainly due to the high prevalence of teenagers of 'other European country' background (90%).

#### Substance use: cannabis use disorder

The adolescent had to have a cannabis use disorder to be eligible for the trial. Most youth qualified for the diagnosis 'cannabis dependence' (84% across sites) and the others (16%) for the diagnosis 'cannabis abuse'.

The rate of cannabis dependence differed between sites (*χ*^2 ^[4, 450] = 20.6, *p *< 0.001), being lowest in The Hague and Paris, where approximately 25% presented with the milder diagnosis 'abuse'.

#### Substance use: alcohol use disorder

Alcohol abuse and alcohol dependence are combined as 'alcohol use disorders' in Table 2. Sites varied in prevalence of alcohol use disorders (*χ*^2 ^[4, 450] = 68.0, *p *< 0.001). These disorders were common, except in The Hague (16%) and Paris (30%).

#### Substance use: other drugs

Many adolescents had experience with other drugs, but not on a regular basis. For no class of drugs other than cannabis, substance use disorder rate exceeded the 5% level at any site.

#### Other problem behaviour: legal problems

Across sites, 34% of the adolescents had been arrested once or more in the 90 days preceding the baseline assessment (Table 2). Sites differed on this measure (*χ*^2 ^[4, 447] = 22.7, *p *< 0.001), with arrest rate being lowest in Berlin (20%) and The Hague (28%) and highest in Geneva (50%).

More than one reason of arrest could be listed per case. Arrests were mostly for drug offenses, property crimes and violence, but this differed between sites. Of the arrested youth in Brussels, 23% were charged for a drug offense and in Paris and Geneva close to 40%. The figure for Berlin was 8% and for The Hague 1%. Other reasons of arrest varied between sites as well. Of Swiss arrested adolescents, 30% had been booked for property crimes, as compared with approximately 10% of Belgian, German and Dutch teenagers, and with a low of 2% in Paris. Violent crimes accounted for 8% - 20% of adolescents who had been arrested, with Paris again being the lowest ranking site (2%).

#### Risk factor: mental and behavioural co-morbidity

Co-morbidity is a risk factor for substance use disorders and other problem behaviours [17]. Sites varied in adolescent YSR self-report of externalizing (aggression and delinquent behaviour) but not clearly in internalizing problems (anxiety and depression). Externalizing problems: *F *[4, 425] = 6.4, *p *< 0.001. Internalizing problems: ANOVA, *F *[4, 425] = 2.3, *p *= 0.06. Youth in Berlin reported higher levels of externalizing symptoms than youth in The Hague and Paris, and youth in Geneva reported higher levels than youth in Paris (Table 2).

Most adolescents had not received any mental health or behavioural treatment or other professional intervention in the 90 days before baseline assessment. The sites did not differ on this measure.

#### Social risk factors

Social risk factors influencing substance misuse and other problem behaviour are hanging out with antisocial rather than with pro-social peers, and poor rooting in school or work [17].

Across sites, 88% of the adolescents said they had one or more friends with a drug problem (ranging from 79% in Berlin to 99% in The Hague (*χ*^2 ^[4, 449] = 23.5, *p *< 0.001). Contact with alcohol misusing peers also varied between sites (*χ*^2 ^[4, 449] = 68.1, *p *< 0.001), and was most frequent in The Hague (99%) and lowest in Brussels (52%). Having delinquent friends was highest in The Hague (95%) and lowest in Paris (62%), with a significant difference across sites (*χ*^2 ^[4, 449] = 37.1, *p *< 0.001).

Most adolescents were still in school (Table 2), though more so in Brussels and Paris than at the other sites (*χ*^2 ^[4, 446] = 14.7, *p *= 0.005). Lower and middle education prevailed in Berlin (56%), Brussels (63%), The Hague (70%), and Paris (63%). This figure was lowest in Geneva (47%).

Having a job and pursuing employment varied across sites. We took together the youth with a regular and with a temporary job (Table 2). Sites differed on this combined measure (*χ*^2 ^[8, 438] = 158.4, *p *< 0.001), with those having paid work (mostly jobs on the side) being most prevalent in Brussels and The Hague and least in Paris. Of the French adolescents, 91% said they were not looking for paid work - much more than at the other sites. The Hague scored lowest on this measure, with 18%.

### Cross-site comparisons on other baseline characteristics: parents

Parents of 39% of all families were still together, but most so in Paris (53% of families) and least in Berlin (28%) (*χ*^2 ^[4, 446] = 18.1, *p *= 0.02). Table 3 shows the mirror image, i.e., the proportion of parents who were separated or divorced - lowest in Paris and highest in Berlin.

**Table 3:** Baseline data about and provided by parents by site and treatment condition

**Variable**	***Brussels***		***Paris***		***Berlin***		***The Hague***		***Geneva***	
	***MDFT***	***TAU***	***MDFT***	***TAU***	***MDFT***	***TAU***	***MDFT***	***TAU***	***MDFT***	***TAU***
Parents divorced/ separated	50%	63%	47%	35%	71%	59%	60%	56%	63%	53%
Parents with substance use/mental problems	27%	33%	16%	18%	47%	36%	29%	26%	33%	33%
Parents with legal problems	27%	27%	5%	5%	16%	16%	14%	11%	10%	13%
Mean internalizing symptoms	22.1 (11.7)	22.0 (12.6)	17.7 (8.3)	20.3 (11.3)	22.0 (10.9)	23.5 (10.3)	17.7 (9.5)	18.1 (10.6)	21.8 (10.4)	21.9 (11.5)
Mean externalizing symptoms	27.4 (10.3)	25.8 (10.7)	23.3 (9.6)	20.6 (10.6)	27.3 (12.7)	25.6 (10.9)	24.6 (12.8)	22.4 (11.8)	29.5 (13.1)	26.8 (13.4)

Figures between brackets = standard deviations

We asked the adolescents to report on problems experienced by their parents and siblings. Overall, about 30% of the youth stated that one or both parents had mental health or addiction problems, with the highest proportion being noted in Berlin (41%) and the lowest in Paris (17%). Alcohol problems were the most prevalent of the three issues surveyed (alcohol, drugs, and mental health). The parent having the problem differed across sites, with fathers being more prevalent as 'problem owner' in Brussels, Geneva, and The Hague, and mothers in Berlin and Paris. A smaller proportion of parents reportedly had a history of legal problems (14%), with Paris at the bottom of the list (5%). Parents with a history of legal problems significantly differed across sites (*χ*^2 ^[4, 450] = 16.0, *p *= 0.003).

As to parent reports of the problems of their children, sites differed on the scores for both internalizing and externalizing symptoms. Internalizing CBCL: *F *[4, 428] = 3.9, *p *= 0.005. Externalizing CBCL: *F *[4, 429] = 4.24, *p *= 0.002. Parents of French youth reported significantly lower levels of externalizing symptoms for their children than parents of Swiss youth, and parents of Dutch youth reported lower levels of internalizing symptoms than parents of German youth.

### Explaining differences between sites: referral source

One would expect randomization to render study conditions comparable on baseline characteristics except for some chance variation. Indeed, this is the case in most single-site trials. However, in multisite trials participant demographics and clinical characteristics, as well as treatment effectiveness, may vary across sites [7,8]. In INCANT too, we saw cross-site differences in baseline demographic and clinical characteristics, though not between treatment conditions. We now turn to attempts we made to account for these cross-site differences.

The general treatment culture varies between the five INCANT countries because of differences in norms, social structures, and local and national policies. Such differences will not disappear through randomization. We assumed that 'referral source' might be a good proxy for the factors underlying heterogeneity between sites.

As seen in Table 4 referral source varied across sites (*χ*^2 ^[16, 450] = 466.5, *p *< 0.001). Self-referral and referral by family and friends were more common in Brussels and Paris than in Berlin and The Hague, where referral by other treatment and care agencies carried more weight, and in Geneva with its high proportion of referral by Justice-related institutions.

**Table 4:** Source of referral of adolescents per site

**Site**	***Self-referred***	***Relatives, friends***	***School***	***Treatment and care agencies***	***Justice***
Brussels	5%	62%	3%	7%	23%
Paris	14%	59%	10%	11%	6%
Berlin	5%	17%	1%	75%	2%
The Hague	2%	6%	0%	73%	19%
Geneva	2%	16%	2%	8%	72%
**TOTAL**	6%	30%	3%	42%	19%

#### Coercion

In Table 5 referral source has been classified as either Self-Determined (SD) or Externally Coerced (EC). Across sites, 49% of referrals were SD and 51% EC. SD dominated in Brussels and Paris, but not in The Hague and Geneva, where EC prevailed. In Berlin, SD and EC matched each other in frequency. The across-sites differences were statistically significant (*χ*^2 ^[4, 450] = 167.1, *p *< 0.001).

**Table 5:** Referral by degree of coercion, site and treatment condition

**Referral type**	***Brussels***		***Paris***		***Berlin***		***The Hague***		***Geneva***	
	***MDFT***	***TAU***	***MDFT***	***TAU***	***MDFT***	***TAU***	***MDFT***	***TAU***	***MDFT***	***TAU***
Self-Determined	70%	77%	92%	94%	48%	44%	6%	17%	20%	33%
Externally Coerced	30%	23%	8%	6%	52%	56%	94%	83%	80%	67%

Referral source and SD/EC distinction were similar for treatment conditions (MDFT and TAU) at all sites (*p *> 0.7).

When referral source was taken into account, quite a few initial differences in the baseline variables to be used as outcome measures (substance use, alcohol use disorder, co-morbid internalizing mental health symptoms) and in demographic characteristics (type of school attended) were no longer significant.

However, accounting for referral source did not fully redress differences in adolescent reports of externalizing symptoms (*F *[4, 430] = 2.6, *p *= 0.04). Berlin youth reported more externalizing symptoms than youth in The Hague and Paris did, and youth in Geneva reported more than youth in Paris.

We also performed a multivariate analysis of variance (MANOVA) for three intended outcome measures (TLFB, YSR, and CBCL) together, to examine the extent to which referral source accounted for site differences when considering variables combined. The result of the multivariate test was significant (*F *[20, 1350] = 1.77, *p *= 0.02). Subsequent univariate tests showed that, in addition to the variable 'externalizing symptoms' just mentioned, sites also differed in frequency of adolescent substance use (*F *[4, 411] = 2.76, *p *= 0.03) when the TLFB, YSR and CBCL measures were considered together, with Swiss youth reporting significantly less substance use than adolescents at the other sites.

## Discussion and Conclusion

INCANT is a transnational trial involving sites in Berlin, Brussels, Geneva, The Hague and Paris. *Within *sites, MDFT and TAU groups were similar on virtually all baseline characteristics studied, including stratification variables and referral source. In contrast, baseline characteristics of the participants substantially differed *across *INCANT sites.

### Referral source

In major part, the between-sites differences could be explained by referral policy. In Brussels and Paris, most adolescents sought treatment themselves or through some non-coercive encouragement by family, friends or sometimes school, whereas referral was more coercive in Berlin and even more so in Geneva and The Hague. When correcting for self-determined or coercive nature of referral, most between-site differences disappeared.

Why did referral type differ? This was partly due to the selection of sites. For instance, we wanted to have a site in the inner-city of Paris. But there, families are affluent, housing is expensive - to such extent that it protects against divorce of parents -, and schools are so strict that pupils are being sent off if failing one class. Under those circumstances, youth have school-related and other personal motives to seek help. In Brussels, there was no professional referral to adolescent substance use treatment to speak off when we started INCANT. So, the Belgians advertised treatment through media channels, resulting in a high rate of self- or family-referral. In Berlin, INCANT site Therapieladen reinforced its network of local treatment and care agencies. Here, many referrals were from sheltered living facilities (Betreutes Wohnen). In Geneva and The Hague, detention and other justice-imposed measures are common and this was reflected in a higher rate of coerced referral. This is not to say that, for instance, the Dutch adolescents were more delinquent or more strongly disordered than at the other sites. Detaining youth is much more common in the Netherlands (and Switzerland) than in the other INCANT countries [18]. The dominant presence of justice-related authorities dictates referral practice.

### Profiles

Study site populations were similar in many respects, but nevertheless had distinct profiles. Take the example of The Hague. The Dutch teenagers more often had friends with a substance use or criminal behaviour problems than at the other sites. Still, when examining overall clinical severity, the Dutch adolescents were not as impaired as youth at some other sites. The rate of cannabis dependence and the rate of alcohol use disorders were lowest in The Hague and Paris. The frequency of self-reported internalizing and externalizing symptoms, as well as arrest rates, were also low. Most Dutch teenagers had an income from a job or other employment, which may have kept them engaged in activities that competed with time that could otherwise have been spent using drugs or associating with delinquent peers.

The adolescent study population in Paris also presented with less impairment than youth in Berlin, Geneva, and Brussels. Cannabis dependence and alcohol use disorder rates were relatively low among French youth, as were the rates of externalizing symptoms and arrests. Most teenagers of the Paris site were not looking for work, but were well provided for by their affluent families.

The populations in Berlin, Brussels and Geneva scored higher than those from The Hague and Paris on cannabis dependence, alcohol use disorders, and externalizing symptoms. Parent divorce/separation rate was highest in Berlin; the proportion of parents with legal problems was highest in Brussels. Recent justice involvement was highest among Geneva youth.

### Sites are not the same as countries

We compared sites from five European countries. The data collected pertain to these sites, but not necessarily to the country where a site was located. What is true in Berlin or Paris, for instance, may not be true in all of Germany or France, respectively. A city does not represent the countryside. One site in a (semi-)federal state, such as Belgium, Germany and Switzerland, does not stand for the country as a whole. Therefore, all conclusions in this paper are restricted to the sites selected for INCANT, so do not extend to other sites.

### The demands of a multisite trial

When five European governments are interested in having a treatment tested for adolescents with cannabis use disorder, as in our case, it would not be efficient to carry out five separate trials. A multisite trial has the advantage of having increased statistical power, and of being more relevant for practice (external validity) than a stringently controlled local trial would be. However, a multisite trial also carries risks, the most important one being lack of equivalence between local study populations. Our site populations differed in many respects.

Variations between local study populations are not an insurmountable barrier for pooling data across sites, if they somehow can be accounted for [7,8]. This appeared to be possible in INCANT. The major explanation of the cross-site variations in baseline characteristics was referral source. Correcting for referral source made most differences disappear. Therefore, we will use referral source as covariate in future cross-site INCANT outcome analyses.

An alternative approach for dealing with multisite issues may be treating site as a random effect (i.e., a variance component accounting for cross-site variation in outcome [19]). However, this would require a larger number of study sites than the five in INCANT [20].

Our experience with a transnational trial in an area with such a limited history of experimental studies was satisfactory. It appeared possible to include sites from different European countries, with different treatment systems and policies, into one meaningful cross-national RCT. We gained more insight into the applicability of MDFT in practice than would have been possible in stand-alone studies. As next papers will show, the cross-national study design did not stand in the way of demonstrating the effectiveness of MDFT; on the contrary. Multisite trials are a recommendable option, provided each site recruits a sufficient number of cases to allow for local analyses if sites appear to differ on unexplainable grounds.

## Abbreviations

ADI: Adolescent Diagnostic Interview; ANOVA: analysis of variance; CBCL: Child Behavior Checklist; CEDAT: Conseils Aide et Action contre le Toximanie; CTRADA: Center for Treatment Research on Adolescent Drug Abuse; EC: externally coerced referral; INCANT: International Cannabis Need of Treatment study; IQ: intelligence quotient; MANOVA: multivariate analysis of variance; MDFT: Multidimensional Family Therapy; Sd: standard deviation; SD: self-determined referral; TAU: Treatment As Usual; TAU-e: manualized TAU in France (e = 'explicit'); TLFB: Timeline Follow-Back; YSR: Youth Self Report

## Competing interests

All authors declare that they have no competing interests.

## Authors' contributions

OP, GH and HR were substantially involved in the conception of the study. HR designed and coordinated the overall study, with statistical advice from GH, whereas OP set up and directed the study in Paris. OP, GH, and HR all had a major part in the statistical analyses and the interpretation of the results, and in drafting the present publication. CS, MT, RR, TA and PW helped in recruiting study participants, administered the questionnaires and interviews at baseline and follow-up, guarded follow-up completion rates, and entered all data into the INCANT database. All have approved the present publication.

## Pre-publication history

The pre-publication history for this paper can be accessed here:

http://www.biomedcentral.com/1471-244X/11/110/prepub
